# Validity of the SNAP-IV For ADHD Assessment in South African Children With Neurodevelopmental Disorders

**DOI:** 10.1007/s10803-022-05530-1

**Published:** 2022-04-22

**Authors:** Michal R. Zieff, Michelle Hoogenhout, Emma Eastman, Björn U. Christ, Alice Galvin, Victoria de Menil, Amina Abubakar, Charles R. Newton, Elise Robinson, Kirsten A. Donald

**Affiliations:** 1 Department of Paediatrics and Child Health, University of Cape Town, Red Cross War Memorial Children’s Hospital, Klipfontein Road, 7700 Rondebosch, Cape Town, South Africa; 2 Stanley Centre for Psychiatric Research, Broad Institute of MIT and Harvard, 75 Ames Street, 02142 Cambridge, MA USA; 3 Department of Epidemiology, Harvard School of Public Health, 677 Huntington Avenue, 02115 Boston, MA USA; 4 Neurosciences Unit, Clinical Department, KEMRI-Wellcome Trust Collaborative Research Programme, PO Box 230-80108, Kilifi, Kenya; 5 Department of Psychiatry, University of Oxford, Warneford Hospital, OX3 7JX Oxford, UK; 6 Institute for Human Development, Aga Khan University, P.O. Box 30270-00100, Nairobi, Kenya

**Keywords:** SNAP-IV, Attention-Deficit/Hyperactivity disorder, Neurodevelopmental disorders, Psychometric, Behavior rating scale

## Abstract

This study investigated the psychometric properties of the Swanson, Nolan, and Pelham ADHD Rating Scale (SNAP-IV) in a sample of South African children with neurodevelopmental disorders (*n* = 201), primarily Autism Spectrum Disorder and Intellectual Disability. We conducted a confirmatory factor analysis to inspect the two-factor structure of the SNAP-IV. We also calculated ordinal coefficient alpha to estimate internal consistency. Fit statistics for the two-factor model approached acceptable levels. The model fit improved slightly after removing an item related to spoken language. The subscales had acceptable internal consistencies. Findings partially support the use of the SNAP-IV in this group of children. However, there are limitations to its performance in this population likely related to the presence of neurodevelopmental disorders.

Behavioral rating scales are often used in clinical settings to evaluate symptoms of Attention-Deficit/Hyperactivity Disorder (ADHD) in children and adolescents. Data derived from ADHD rating scales may play an important supportive role in clinical assessment. For example, clinicians may rely on rating scales to quantify the severity of impairments, to monitor changes in behavior over time or monitor impact of interventions (Young et al., 2020). It is therefore important that the information derived from these measures is valid and reliable.

ADHD frequently occurs as a comorbidity in children with other neurodevelopmental disorders (NDDs), including Autism Spectrum Disorder (ASD), Intellectual Disability (ID), and Specific Learning Disorders (SLD). Clinically significant ADHD symptoms may be present in approximately 28–78% of children with ASD, 23% of children with ID, and 40% of children with SLDs (Bandla et al., 2017; Gadow et al., 2005; Lai et al., 2019; Lee & Ousley, 2006; Leyfer et al., 2006; Neece et al., 2011; Rao & Landa, 2014; Sinzig et al., 2009; Smith & Adams, 2006; Stevens et al., 2016). Notwithstanding the frequent co-occurrence of ADHD and other NDDs, less in known about the nature of ADHD symptoms in children with other NDDs than about ADHD symptoms in children who are otherwise typically developing (Reilly & Holland, 2011). A likely consequence of our limited understanding of ADHD in this population is that children with other NDDs are frequently excluded from normative and validation samples of behavioral rating scales designed to evaluate symptoms of ADHD (Antshel et al., 2006). This, in turn, makes it difficult to assess whether our approach to measuring ADHD-related behaviors in children with other NDDs is valid. The result is a lack of evidence demonstrating that ADHD rating scales measure the same underlying constructs (i.e., have construct validity) with children who have other NDDs.

There is some evidence suggesting that ADHD rating scales may not be valid for children with ASD. One study evaluating the ADHD Rating Scale-Fourth Edition in a sample of children with ASD (without comorbid ID) found that the scale did not adequately distinguish between inattention and hyperactivity-impulsivity, the two constructs thought to underpin ADHD, in this group (Yerys et al., 2017). A confirmatory factor analysis (CFA) found three items intended to measure inattention, including “Does not listen when spoken to directly” and “Easily distracted”, were also associated with the hyperactivity-impulsivity factor in these children. These results suggest that the presence of comorbid ASD may influence the ratings of target (ADHD) behaviors. However, in terms of the overall phenotype described, higher ADHD scores were associated with higher levels of externalizing behaviors (e.g., aggression) relative to internalizing behaviors (e.g., depression), resembling findings from typically developing samples (Reiersen & Todorov, 2013).

One frequently used ADHD rating scale is the Swanson, Nolan, and Pelham ADHD Rating Scale (4th edition; SNAP-IV; Swanson et al., 2012). In research contexts, the SNAP-IV is typically used with school-based, non-clinical samples (Bussing et al., 2008). However, a few promising studies support the use of the SNAP-IV with children who have ID. Two related studies found that the SNAP-IV subscales had strong psychometric properties in a sample of children with ID. The SNAP-IV had excellent reliability and concurrent validity, demonstrated by large, positive correlations with scores on other ADHD rating scales (Miller, Fee, & Jones, 2004; Miller, Fee, & Netterville, 2004). However, these studies did not investigate the structural validity of the SNAP-IV.

In clinical contexts, the SNAP-IV is sometimes used to aid in the assessment of ADHD in child patients with other NDDs. The SNAP-IV is used clinically in this way in the Western Cape province of South Africa, where, similar to other resource-limited settings, specialist neurodevelopmental services accessible to the majority of the population are frequently over-burdened. In addition, the clinical severity of neurodevelopmental populations presenting to tertiary hospitals in Africa is typically high, resulting in a large number of non-verbal children (Bakare & Munir, 2011). Rating scales are especially useful for assessment purposes in low-resourced clinical settings as they are quick and inexpensive to administer. However, until there is sufficient psychometric evidence to support use in these contexts, results should be interpreted with caution. To the best of our knowledge, there are no published studies to date that investigate the psychometric properties of the SNAP-IV in a sample of children with NDDs other than ID.

The aim of this study was to evaluate the validity of scores derived from the SNAP-IV in a sample of young South African children with NDDs. We used the analyses conducted by Yerys and colleagues (2017) as a template for the current study. We hypothesized that (i) the SNAP-IV items would measure two distinguishable constructs, namely, inattention and hyperactivity-impulsivity, (ii) the SNAP-IV subscale scores would correlate positively with subscale scores of another measuring ADHD-related behaviors, and (iii) the SNAP-IV would have strong, positive correlations with externalizing behaviors relative to internalizing behaviors.

## Methods

### Participants

**Fig. 1:**
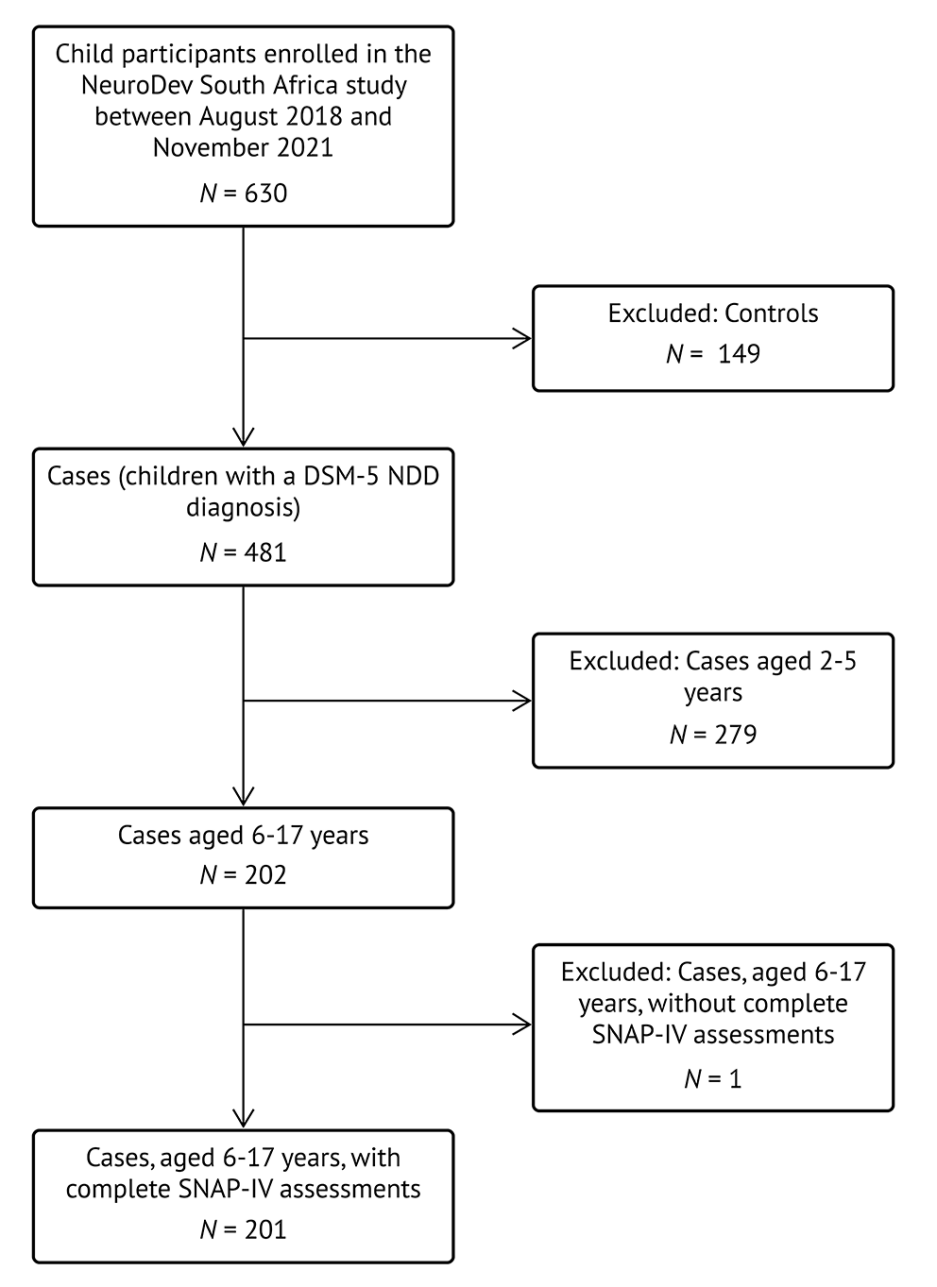
Sample Selection for the Current Study Within the NeuroDEV South Africa Study. (Note. DSM-5 = Diagnostic and Statistical Manual of Mental Disorders, 5th edition. NDD = Neurodevelopmental disorder. SNAP-IV = SNAP-IV = Swanson, Nolan, and Pelham ADHD Rating Scale, 4th edition.)

This study was embedded within a larger project titled “NeuroDEV South Africa”, which administers the SNAP-IV to parents of case children (children with NDDs) aged 6–17 years (de Menil et al., 2019). This large-scale, multi-site study aims to investigate the genetic architecture and phenotypic manifestations of NDDs in African populations. Figure 1 outlines the sample selection process for the current study within the context of the larger NeuroDEV South Africa study.

We invited parents of children attending outpatient developmental, genetic, speech, and neurology clinics at two tertiary hospitals in Cape Town to participate in the NeuroDEV South Africa study. Children were eligible for inclusion as cases if they had a confirmed diagnosis of one or more of the following Diagnostic and Statistical Manual of Mental Disorders 5th edition (DSM-5) NDDs: ADHD, ASD, SLD, ID or Communication Disorder (CD; American Psychiatric Association, 2013). Children with a primary neuromotor disability (e.g., cerebral palsy) were not eligible to participate. We analyzed data from all case children aged 6 years and older who were enrolled in the NeuroDEV South Africa study between August 2018 and November 2021, except for one participant who did not complete the SNAP-IV.

### Measures

#### Demographics Questionnaire and Asset Index

Parents completed a demographic questionnaire, which included questions about the child and parents’ home languages. The Asset Index is a socio-demographic questionnaire adapted from previous South African studies, including questions regarding parental educational attainment and household income (Myer et al., 2008; Stein et al., 2015).

#### Swanson, Nolan and Pelham Questionnaire ADHD Rating Scale (SNAP-IV; Parent Form)

The SNAP-IV ADHD rating scale is an 18-item self-report questionnaire designed to measure symptoms of ADHD (Swanson et al., 2012). The items are consistent with the DSM-5 ADHD diagnostic criteria and are designed to distinguish between different symptom presentations of ADHD, namely, inattentive, hyperactive-impulsive, and combined (both inattentive and hyperactive/impulsive). The subscales are named accordingly; ‘Inattention’ (IN, 9 items) and ‘Hyperactivity-Impulsivity’ (HI, 9 items). The items are presented as DSM-5 symptoms (e.g., “Often is forgetful in daily activities”). For each item, respondents select one of four response options (0 “Not at all”, 1 “Just a little”, 2 “Quite a bit”, or 3 “Very much”) that best describes the child’s behavior over the past year. Subscale and total scores are calculated as an average score across relevant items. We included a “not applicable” (N/A) response option to capture questions relating to speech in non-verbal children.

We obtained Afrikaans and Xhosa translations of the SNAP-IV from another South African research group that had previously used the SNAP-IV (Zeegers et al., 2010). Two Afrikaans and three Xhosa-speaking members of the NeuroDEV South Africa research team independently reviewed the Afrikaans and Xhosa translations respectively to confirm that the translations were tapping into the intended constructs. The Afrikaans reviewed team consisted of a senior research assistant with a neuropsychology background (B.U.C) and a pediatric research nurse. The Xhosa review team comprised two research assistants with a psychology background (including M.R.Z), and a pediatric research nurse. Each team consolidated their suggestions for revisions (minor revisions to the wording of items) to create the final translation versions.

To estimate the prevalence of clinically significant ADHD symptoms in this sample, we set cut-off scores that were aligned with the DSM-5 diagnostic criteria for ADHD. The DSM-5 requires an individual to exhibit at least 6 symptoms in at least one domain (inattention or hyperactivity-impulsivity) to qualify for a diagnosis of ADHD. Pelham and colleagues (1992) recommend defining the presence of a symptom by a 3-point score on the SNAP-IV items, as it produces prevalence rates similar to those reported in the general population. Therefore, we estimated the prevalence of ADHD symptomology in the sample by calculating the number of participants who scored a ‘3’ on at least six items in at least one domain.

#### Child Behavior Checklist for Ages 6–18 (CBCL/6–18)

The CBCL/6–18 is a self-report questionnaire designed to assess specific problematic behaviors in school-age children, as reported by caregivers (Achenbach & Rescorla, 2001). It is considered a “gold standard” tool for assessing behavioral problems in children and has been validated in over 30 countries (Ivanova et al., 2007). The items listed in the CBCL/6–18 are designed to align with the DSM-5 diagnostic criteria for a number of behavioral disorders, including ADHD. This study was concerned with four subscales: The Attention Problems syndrome scale (10 items), the ADHD DSM-oriented scale (7 items), the Aggressive Behavior syndrome scale (18 items), and the Withdrawn/Depressed syndrome scale (8 items). We used the latter two subscales as measures of externalizing and internalizing behaviors respectively. We obtained licenses to administer the ASEBA Afrikaans and Xhosa translations of the CBCL/6–18. For each item, parents rated the frequency/severity of their child’s behavior in the past six months using a three-point scale; 0 (‘not true’), 1 (‘somewhat or sometimes true’), or 2 (‘very or often true’). Subscale scores were calculated by summing the scores of all relevant items. The CBCL/6–18 is widely used in research with children who have ASD. There is some evidence to support the subscales’ validity and reliability in this population (Dovgan et al., 2019; Pandolfi et al., 2012, 2014).

### Procedure

We obtained informed consent from parents of child participants. If developmentally appropriate, we obtained assent from child participants over the age of 12 years. A member of the data collection team (comprising medical doctors, research nurses, and psychologists) verbally administered the SNAP-IV to all parents as well as the CBCL/6–18 subscales to a subset of parents, in their preferred language. Administration of the CBCL/6–18 began in May 2019, nine months after data collection for the umbrella study commenced. Although we did not record the language of administration for specific tools, anecdotal reports indicate that the majority of participants elected to complete the questionnaires in English.

The University of Cape Town’s Human Research Ethics Committee approved this research (367/2019) as part of the NeuroDEV South Africa study (810/2016). The NeuroDEV study also received ethics approval from the Harvard T. H. Chan School of Public Health Institutional Review Board (17-1260) and the Western Cape Department of Health, South Africa.

### Statistical analyses

We performed all statistical analyses in RStudio (Version 1.3.1093) for R (Version 4.0.2; R Core Team, 2019) We used the ‘psych’ package to conduct exploratory data analysis (Revelle, 2020). To estimate the concurrent validity, convergent validity, and discriminant validity of the SNAP-IV, we computed Pearson’s correlation coefficients between SNAP-IV subscale scores and CBCL/6–18 subscale scores, and the ‘corcor’ package to statistically compare correlation coefficients (Diedenhofen & Musch, 2015). We estimated the internal consistency reliability of the SNAP-IV subscales using ordinal coefficient alpha (Zumbo et al., 2007). To test the expected two-factor structure of the SNAP-IV, we conducted a CFA using the ‘lavaan’ package (Version 0.6-5; Rosseel, 2012). CFA examines relationships between observed variables (indicators) and latent variables (factors). CFA is used to confirm an a priori specification (i.e., hypothesis) about the underlying structure of a tool (Kline, 2016). We used global and local fit statistics to determine the usefulness of the model (Brown, 2015). The chi square statistic (χ^2^) is a test of exact fit between the model and the data. The Tucker-Lewis Index (TLI) conveys information about the “goodness of fit” between the model and the data. A higher value indicates better fit, and should ideally exceed 0.95 (i.e., the specified model should improve the fit by 95% relative to no model). The Root Mean Square Error of Approximation (RMSEA) is a measure of approximate fit, where 0 indicates a perfect fit and 0.05 indicates close fit. The Standardised Root Mean Square Residual (SRMR) represents the average difference between the observed and predicted correlations matrices (i.e., the average residual correlation). Like the RMSEA, SRMR values close to 0 suggest good fit. Modification Indices (MIs) approximate the amount by which chi-square value will decrease (i.e., model fit will improve) if an unspecified parameter were to be estimated. Parameters with MIs greater than 3.84 indicate a statistically significant decrease in the chi-square statistic, or improved model fit. Each MI is associated with a standardized expected parameter change (SEPC), the magnitude and direction of which approximates how much the parameter is expected to change if it were to be estimated. Standardised coefficients (also known as ‘factor loadings’) are estimates of direct effects between latent and observed variables. The squared factor loadings indicate the proportion of variance in each observed variable that is explained by the latent factor.

Given our chosen statistical analyses, we conducted a priori power calculations to determine the minimum required sample sizes to obtain reasonable power in our two main analyses. Using the ‘semPower’ package in R, we determined that obtain a RMSEA of 0.05 with 134 degrees of freedom and powers of 80% and 90% respectively, sample sizes of 139 and 168 are required (Moshagen & Erdfelder, 2016). For the correlation, an a priori power analysis using G*Power software indicated that to achieve an effect size of 0.30 (conservatively selected) for a one-tailed test (α = 0.05) with 80% and 90% powers respectively, the number of required observations are 64 and 88 respectively (Faul et al., 2007).

## Results

The sample included 201 child participants aged 6–17 years (*M* = 8.16, *SD* = 2.61). Table 1 presents the sociodemographic characteristics of the sample, as well as information related to DSM-5 diagnoses, level of spoken language, and highest level of education. Most children came from low- and middle-income families that spoke one of the three main languages spoken in the Western Cape of South Africa. The predominance of male participants (> 70%) is typical of study samples comprising children with NDDs (Springer et al., 2013).

Seventy-five child participants (37%) had more than one NDD diagnosis. The most frequent diagnoses were ID and ASD. ASD and ID were comorbid in 49 participants (24% of the sample). In this sample, CD was primarily comorbid with ID, with only five children having CD a primary diagnosis. Twenty-two children (11%) were diagnosed with ADHD, which in all but two participants was comorbid with at least one other NDD, including ASD (*n* = 4), ID (*n* = 11), ASD and ID (*n* = 3), CD (*n* = 2), or SLD (*n* = 1). Figure S1 in the supplement is a Venn diagram displaying overlap between clinical DSM-5 diagnoses. One hundred and twenty-one children (60%) had delayed (i.e., non-fluent) speech. Sixty-six (33%) children were not enrolled in a formal schooling system.

Table 2 presents a summary of the sample’s SNAP-IV scores, as well as item response frequencies. Figures S2 and S3 in the supplement display the distribution of subscale scores. Using our tentative cutoff scores for the SNAP-IV, 69 children (34%) exhibited clinically significant symptoms of ADHD. Twenty-five children (12%) met the cut-off for a predominantly inattentive presentation of ADHD, 33 (16%) for a predominantly hyperactive/impulsive presentation, and 11 (5%) for a combined presentation. Of the 22 participants with a confirmed ADHD diagnosis, 3 (14%) met the criterion for a predominantly inattentive presentation, 4 (18%) for a predominantly hyperactive/impulsive presentation, and 5 (23%) for a combined presentation. On average, respondents strongly endorsed the SNAP-IV items, with percentages of “quite a bit” or “very much” responses ranging from 22 to 72% (*M* = 57.40, *SD* = 13.55) and percentages of “very much” responses ranging from 11 to 55% (*M* = 35.88, *SD* = 11.04). Response patterns did not differ substantially by primary diagnosis (see Figure S3 in the supplement). Eleven items had small proportions of N/A responses, while Items 15 (“Talks excessively”) and 16 (“Blurts out answers”) had 51 (25%) and 61 (30%) N/A responses respectively.

**Table 1:** Sociodemographic and Diagnostic Information (*N *= 201)

Variable	Frequency (%)	
**Sex**		
Male	150	(74.62)
Female	51	(25.37)
**Home language**		
English	115	(57.21)
Afrikaans	23	(11.44)
Xhosa	55	(27.36)
Other	8	(3.98)
**Asset Index**		
** Monthly household income**		
< $66	7	(3.48)
$66 –$333	77	(38.31)
$333 – $666	54	(26.87)
$666 – $1000	28	(13.93)
> $1000	28	(13.93)
Unknown	7	(3.48)
** Highest level of maternal education**		
No education	1	(0.50)
Primary school	9	(4.48)
High school without completion	71	(35.32)
Completed high school	55	(27.36)
Tertiary education (partial or complete)	64	(31.84)
Unknown	1	(0.50)
Overall clinical DSM-5 diagnoses (including comorbid diagnoses)*		
Attention-Deficit/Hyperactivity Disorder	22	(10.95)
Autism Spectrum Disorders	111	(55.22)
Intellectual Disability	130	(64.68)
Communication Disorders	19	(9.45)
Specific Learning Disorders	3	(1.49)
**Language level**		
No spoken language	37	(18.41)
Single words only	30	(14.93)
Phrases	54	(26.87)
Fluent	80	(39.80)
**Highest level of education**		
Never attended	35	(17.41)
Crèche	29	(14.43)
Non-academic/alternative curriculum	48	(23.88)
Special needs school	28	(13.93)
Pre-primary school	17	(8.46)
Primary school (Grades 1–7)	41	(20.40)
High school (Grades 8–12)	1	(0.50)
Other	2	(1.00)

*Note.* ‘Other’ home languages included Shona (*n* = 4), Chichewa (*n* = 2), Swahili (*n* = 1), and Lingala (*n* = 1). All participants who spoke the aforementioned ‘other’ languages also spoke English at home. At the time of writing, $1.00 ≈ ZAR15.00. Information about the child’s diagnosis, language level, and education were extracted from the child’s medical records by a medical officer. DSM-5 = Diagnostic and Statistical Manual of Mental Disorders, 5th ed. *Percentages do not add up to 100 as children may have more than one diagnosis.

**Table 2:** SNAP-IV Item Frequencies, Means, and Standard Deviations (N = 201)

SNAP-IV Item		Response frequencies (%)					Percentage responses		*M* (*SD*)
		“0”	“1”	“2”	“3”	“N/A”	% “2” or “3”	% “3”	
Inattention									1.81 (0.65)
1	Fails to give close attention to details	25 (12.44)	57 (28.36)	58 (28.86)	60 (29.85)	1 (0.50)	58.71	29.85	1.76 (1.02)
2	Difficulty sustaining attention	21 (10.45)	35 (17.41)	63 (31.34)	82 (40.80)	0 (0.00)	72.14	40.80	2.02 (1.00)
3	Does not listen when spoken to directly	11 (5.47)	67 (33.33)	67 (33.33)	54 (26.87)	2 (1.00)	60.20	26.87	1.82 (0.90)
4	Fails to finish tasks	17 (8.46)	47 (23.38)	59 (29.35)	76 (37.81)	2 (1.00)	67.16	37.81	1.97 (0.98)
5	Difficulty organizing tasks and activities	42 (20.90)	37 (18.40)	39 (19.40)	79 (39.30)	4 (1.99)	58.71	39.30	1.79 (1.18)
6	Avoids tasks requiring sustained effort	21 (10.45)	34 (16.92)	44 (21.89)	98 (48.76)	4 (1.99)	70.65	48.76	2.11 (1.04)
7	Loses things necessary for tasks	74 (36.82)	35 (17.41)	33 (16.42)	57 (28.36)	2 (1.00)	44.78	28.36	1.37 (1.25)
8	Distracted by extraneous stimuli	34 (16.92)	23 (11.44)	52 (25.87)	92 (45.77)	0 (0.00)	71.64	45.77	2.00 (1.12)
9	Forgetful in daily activities	58 (28.86)	45 (22.39)	47 (23.38)	48 (23.88)	3 (1.49)	47.26	23.88	1.43 (1.15)
Hyperactivity-Impulsivity									1.71 (0.75)
10	Fidgets or squirms	39 (19.40)	32 (15.92)	40 (19.90)	90 (44.78)	0 (0.00)	64.68	44.78	1.90 (1.17)
11	Leaves seat	40 (19.90)	36 (17.91)	43 (21.39)	79 (39.30)	3 (1.49)	60.70	39.30	1.81 (1.17)
12	Runs about or climbs excessively	61 (30.35)	28 (13.93)	35 (17.41)	76 (37.81)	1 (0.50)	55.22	37.81	1.63 (1.27)
13	Difficulty playing quietly	98 (48.76)	31 (15.42)	32 (15.92)	40 (19.90)	0 (0.00)	35.82	19.90	1.07 (1.20)
14	“On the go” as if “driven by a motor”	48 (23.88)	33 (16.42)	37 (18.40)	83 (41.29)	0 (0.00)	59.70	41.29	1.77 (1.22)
15	Talks excessively	22 (10.95)	37 (18.40)	30 (14.92)	61 (30.35)	51 (25.37)	45.27	30.35	1.87 (1.11)
16	Blurts out answers	61 (30.35)	33 (16.42)	23 (11.44)	23 (11.44)	61 (30.35)	22.89	11.44	1.06 (1.12)
17	Difficulty awaiting turn	31 (15.42)	25 (12.44)	33 (16.42)	111 (55.22)	1 (0.50)	71.64	55.22	2.12 (1.14)
18	Interrupts or intrudes on others	33 (16.42)	34 (16.92)	44 (21.89)	89 (44.28)	1 (0.50)	66.17	44.28	1.95 (1.13)

*Note.* Response categories “0”, “1”, “2”, “3” are labelled as “Not at all”, “A little bit”, “Quite a bit”, and “Very much” respectively, while “N/A” indicates “Not applicable” responses. Some wordings of the SNAP-IV items are abbreviated.

Average polychoric correlation coefficients (*ρ*, see Table 3) between items on the Inattention subscale (*M* = 0.35, *SD* = 0.13) and between items on the Hyperactivity-Impulsivity subscale (*M* = 0.40, *SD* = 0.12) respectively were only slightly larger than cross-subscale correlations (*M* = 0.28, *SD* = 0.12), tentatively suggesting a weak distinction between the two constructs.

**Table 3:** Polychoric Correlation Matrix and Item-Total Correlations for the SNAP-IV ADHD Rating Scale (*N *= 201)

	1	2	3	4	5	6	7	8	9	10	11	12	13	14	15	16	17	ITC
Item 1																		0.66
Item 2	0.55																	0.68
Item 3	0.37	0.41																0.51
Item 4	0.47	0.57	0.42															0.62
Item 5	0.54	0.45	0.28	0.50														0.59
Item 6	0.59	0.54	0.41	0.49	0.45													0.65
Item 7	0.36	0.26	0.26	0.27	0.28	0.31												0.41
Item 8	0.14	0.38	0.29	0.21	0.12	0.22	0.18											0.33
Item 9	0.27	0.27	0.19	0.20	0.40	0.29	0.37	0.26										0.43
Item 10	0.40	0.37	0.32	0.38	0.37	0.26	0.29	0.43	0.29									0.62
Item 11	0.48	0.39	0.32	0.47	0.38	0.42	0.46	0.20	0.11	0.50								0.58
Item 12	0.38	0.30	0.50	0.33	0.28	0.24	0.41	0.32	0.29	0.44	0.66							0.67
Item 13	0.44	0.17	0.36	0.25	0.34	0.32	0.27	0.11	0.23	0.46	0.43	0.58						0.54
Item 14	0.22	0.21	0.37	0.15	0.25	0.18	0.32	0.34	0.26	0.57	0.40	0.57	0.45					0.65
Item 15	0.12	0.01	0.28	0.12	0.17	0.04	0.12	0.25	0.20	0.26	0.11	0.27	0.30	0.39				0.42
Item 16	0.11	0.24	0.40	0.18	0.22	0.03	0.07	0.40	0.01	0.32	0.18	0.32	0.14	0.39	0.43			0.45
Item 17	0.31	0.42	0.43	0.29	0.27	0.29	0.18	0.22	0.17	0.45	0.40	0.42	0.42	0.35	0.38	0.30		0.54
Item 18	0.34	0.32	0.47	0.21	0.29	0.31	0.49	0.32	0.23	0.50	0.40	0.40	0.39	0.46	0.24	0.52	0.50	0.59

*Note.* Correlation coefficients within the black borders are cross-subscale correlation coefficients (i.e., between items on the Inattention and Hyperactivity-Impulsivity subscales). Some correlation coefficients are based on *n* < 201 due to non-applicable responses (see Table 2). ITC = Item-total correlation. ITCs were calculated using total subscale scores (Inattention OR Hyperactivity-Impulsivity), not total SNAP-IV scores. For example, ITC for Item 1 represents the Pearson correlation coefficient between Item 1 scores and Inattention subscale scores (minus Item 1).

We conducted a CFA specifying a model (Model 1) with two latent factors, Inattention and Hyperactivity-Impulsivity, each with nine indicators (SNAP-IV Items 1–9 and Items 10–18 respectively), as well as an additional path estimating the covariance between the two latent factors. Table 4 presents details of the model fit and Table 5, the standardized coefficients (factor loadings). The approximate model fit indices (TLI, RMSEA, and SRMR) were approaching acceptable levels. There was a significant correlation of 0.748 (*p* < 0.001) between the two latent factors. In general, the factors explained relatively high proportions of variance within their respective items. An exception was Items 15 (“Talks excessively”), of which Hyperactivity-Impulsivity explained only 16% variance.

**Table 4:** Model Fit Statistics for Confirmatory Factor Analysis Models

Model	χ^2^ (df)		TLI	RMSEA (90% CI)		SRMR
1^a^	268.39	(134) ***	0.907	0.071	(0.058–0.083) **	0.090
2^b^	242.87	(134) ***	0.916	0.071	(0.056–0.085) *	0.089
3^c^	237.33	(118) ***	0.916	0.071	(0.058–0.084) *	0.086

*Note*. TLI = Tucker-Lewis Index, RMSEA = Root Mean Square Error of Approximation, 90% CI = 90% Confidence intervals, SRMR = Standardized Root Mean Square Residual. Both chi-square (χ^2^) and RMSEA significance tests are accept-support tests. Estimate is significant at *p < 0.05, ** *p* < 0.01, ***p < 0.001. All models measure two latent variables, ‘Inattention’ (‘IN’) and ‘Hyperactivity-Impulsivity’ (‘HI’). Covariance between the two latent variables is also specified. Estimation method is robust weighted least squares with polychoric correlations, which is recommended for ordinal data (Li, 2016).

^a^Model 1: All 18 items – IN (9) and HI (9), full sample (*N* = 201)

^b^Model 2: All 18 items – IN (9) and HI (9), subset of sample (*N* = 164)

^c^Model 3: Item15 removed – IN (9) and HI (8), full sample (*N* = 201)

**Table 5:** Communalities and Standardized Coefficients (Factor Loadings) of SNAP-IV Items in Three CFA Models

SNAP-IV Item	Communalities / Factor loadings									
	Model 1 (*N* = 201)			Model 2 (*N* = 164)			Model 3 *(N* = 201*)*			
	*R* ^*2*^	IN	HI	*R* ^*2*^	IN	HI	*R* ^*2*^	IN	HI	
Inattention										
1. Fails to give close attention	0.532	0.730		0.495	0.703		0.534	0.731		
2. Difficulty sustaining attention	0.496	0.704		0.488	0.695		0.503	0.709		
3. Does not listen when spoken to	0.416	0.645		0.425	0.649		0.409	0.640		
4. Fails to finish tasks	0.436	0.660		0.399	0.628		0.438	0.662		
5. Difficulty organizing tasks	0.414	0.643		0.466	0.679		0.413	0.643		
6. Avoids effortful tasks	0.442	0.665		0.393	0.624		0.447	0.669		
7. Loses things	0.297	0.545		0.267	0.514		0.298	0.546		
8. Distracted by extraneous stimuli	0.208	0.456		0.290	0.537		0.201	0.449		
9. Forgetful in daily activities	0.197	0.444		0.179	0.420		0.194	0.440		
Hyperactivity-Impulsivity										
10. Fidgets or squirms	0.526		0.725	0.586		0.766	0.526		0.725	
11. Leaves their seat	0.537		0.732	0.514		0.714	0.548		0.740	
12. Runs about excessively	0.593		0.770	0.587		0.763	0.594		0.771	
13. Difficulty playing quietly	0.419		0.647	0.374		0.611	0.415		0.644	
14. Often “on the go”	0.430		0.656	0.526		0.733	0.417		0.646	
15. Talks excessively	0.157		0.397	0.144		0.389	-		-	
16. Blurts out answers	0.235		0.485	0.224		0.477	0.209		0.457	
17. Difficulty awaiting turn	0.399		0.632	0.448		0.667	0.388		0.623	
18. Interrupts or intrudes on others	0.463		0.680	0.608		0.774	0.462		0.680	

*Note.* SNAP-IV = Swanson, Nolan, and Pelham ADHD Rating Scale, 4th edition, CFA = Confirmatory factor analysis, IN = Inattention subscale, HI = Hyperactivity-Impulsivity subscale. Factor loadings are standardized.

An inspection of the modification indices and a residual correlation matrix revealed several correlated residuals (*r* > absolute value of 0.1, see Figure S5, a residual correlation matrix for Model 1, and Table S1, both in the supplement). For example, Items 15 (“Talks excessively”) and 16 (“Blurts out answers”; MI = 9.96, *r* = 0.24) had a large positive residual correlation, suggesting that the specified model is not fully accounting for the covariance between these two items. MIs suggested possible “cross-loadings” of Items 3 (“Does not listen”; MI = 16.44, SEPC = 0.487) and 8 (“Distracted by extraneous stimuli”, MI = 12.09, SEPC = 0.432) with the Hyperactivity-Impulsivity factor. We compared Model 1 to a nested one-factor model (Model 1a) to determine if variance in the SNAP-IV indicators is better explained by a single latent factor, “ADHD”. However, the Model 1a’s fit was poorer than that of the original two-factor model, χ^2^ (135, *N =* 201) = 341.38, *p* < 0.001, TLI = 0.859, RMSEA = 0.087.

Taken together, these findings indicate that Item 15 (“Talks excessively”) may be a poor item in this sample. We considered that this item’s poor performance may have been due to its irrelevance for the non-verbal children in this sample. Hence, we ran a second model (Model 2) with a subset of participants who had acquired at least phrase speech (*n* = 164). However, Item 15’s factor loading remained relatively low (see Table 5). We then reran Model 1 without Item 15 (Model 3, *N* = 201) which slightly improved the model fit (see Table 4). This final model (Model 3) specified two related latent factors, Inattention and Hyperactivity-Impulsivity, with 9 and 8 indicators respectively (see Figure S6 in the supplement). MIs suggesting possible cross-loadings of Items 3 (“Does not listen”; MI = 14.89, SEPC = 0.487) and 8 (“Distracted”; MI = 10.60, SEPC = 0.424, see Table S1 in the supplement) with Hyperactivity-Impulsivity remained significant. Item 15 was excluded from all further analyses.

Given evidence to support two correlated unidimensional factors, we calculated subscale means as well as internal consistency reliability statistics for the Inattention (*M* = 1.81, *SD* = 0.65), new Hyperactivity-Impulsivity (*M* = 1.69, *SD* = 0.78), and new Combined ADHD (*M* = 1.76, *SD* = 0.64) subscales respectively. The distributions of subscale scores were approximately normal (skewness = -0.36, -0.32, and -0.34 for IN, HI, and C subscales respectively). Ordinal alpha coefficients were good for Inattention (α = 0.83, 95% CI = 0.79–0.86), Hyperactivity-Impulsivity (α = 0.86, 95% CI = 0.83–0.88), and Combined ADHD (α = 0.90, 95% CI = 0.88–0.92) respectively.

Table S2 in the supplement presents a summary of CBCL/6–18 scores for the subset of the sample that completed the CBCL (*n* = 171), and Table S3 summarizes CFA findings for the CBCL/6–18 subscales. Inattention (SNAP-IV) and Attention Problems (CBCL/6–18) scores were moderately correlated (*r* = 0.55, 95% CI 0.44–0.65, *p* < 0.001). Likewise, there was a moderate, positive correlation between scores on the SNAP-IV Combined ADHD subscale and the CBCL/6–18 ADHD DSM-oriented subscale (*r* = 0.66, 95% CI 0.57–0.74, *p* < 0.001). Combined ADHD (SNAP-IV) scores were also significantly correlated with scores on the CBCL/6–18 Aggression Problems subscale (*r* = 0.48, 95% CI = 0.35–0.59, *p* < 0.001), and the CBCL/6–18 Withdrawn/Depressed scores (*r* = 0.17, 95% CI = 0.02–0.31, *p* = 0.025). The difference between the latter two correlation coefficients was significant (*z* = 3.87, *p* < 0.001), demonstrating good convergent and discriminant validity.

## Discussion

The primary aim of the study was to investigate the construct validity of the SNAP-IV in a sample of children with NDDs. A CFA partially supported the claim that the scale measures two factors, Inattention and Hyperactivity-Impulsivity, barring one item that did not seem to measure either factor clearly, as well as two items that “cross-loaded” onto another factor.

Item 15 (“Talks excessively”) had a small factor loading. In this sample, excessive talking was likely an indicator of a child’s verbal abilities, rather than a measure of hyperactive/impulsive behavior. From a statistical perspective, the large number of N/A responses for Item 15, likely due to the substantial proportion of the sample with limited expressive language, may also have contributed to the item’s weak factor loading. We were not able to use multiple imputation techniques to counteract the effects of missing data points, as the data were not missing at random. The large negative residual correlation between Items 15 and 16 may further account for the item’s poor performance in the model. The model did not sufficiently account for the observed variance shared between these two items. It is possible that similar response patterns for these items may have contributed to the unexpectedly large amount of shared variance (i.e., a caregiver of a non-verbal child who responded to Item 15 with N/A likely responded to Item 16 in the same way).

Items 3 (“Does not listen when spoken to directly”) and 8 (“Distracted by extraneous stimuli”) were also consistently problematic across all models. The cross-loadings suggest that these behaviours were not good measures of inattention in this sample. It is possible that parents of children with ASD endorsed these items due to impairments in social communication rather than difficulties with inattention and impulsivity/hyperactivity specifically. For example, the wording of the item “easily distracted by extraneous stimuli” may be interpreted as an impairment of social reciprocity, a core symptom of ASD (e.g., a child not making direct eye contact with the person they are communicating with) or inattentiveness, a core symptom of ADHD (e.g., a child being unable to sustain attention during one activity or another). Misinterpretation of item wordings may result in over-endorsement of items or “false positives”, perhaps explaining the higher-than-expected proportion of children in the sample who met the cutoffs for clinically significant ADHD-related behaviour. This finding highlights the importance of using clinical interview techniques alongside behavioural screening tools to disentangle symptoms that may present similarly, albeit with different underlying causes. Thorough questioning around the context of these ADHD-related behaviours will shed light on whether a symptom is indeed suggestive of ADHD or better explained by another NDD (e.g., ASD).

Moderate, positive relationships between the SNAP-IV and CBCL/6–18 ADHD-related subscales supported the concurrent validity of the SNAP. It is worth noting that the SNAP-IV and CBCL/6–18 are similar in terms of item content and response format. Hence it is possible that the correlation coefficients may have been inflated by common method variance. The SNAP-IV also demonstrated convergent and discriminant validity when correlating ADHD-related behaviors with internalizing and externalizing behaviors respectively. Combined ADHD (SNAP-IV) was more strongly associated with aggressive (externalizing) behaviour than with withdrawn/depressed (internalizing) behavior, as measured by the CBCL/6–18. It is likely, though, that sample-related factors affected responses to the CBCL/6–18. For example, items such as “Teases a lot”, “Threatens people” (Aggressive Behavior), “Refuses to talk” and “Secretive” (Withdrawn/Depressed) were not applicable to children who were non-verbal. Perhaps the pre-school version of the CBCL, the CBCL/1.5-5 may have been a more appropriate measure of internalizing and externalizing behaviors, given the average developmental level of the children in this sample.

Most children in the current sample with a confirmed ADHD diagnosis did not meet the SNAP-IV cutoff for clinically significant ADHD-related behaviours. The DSM-5 requires that symptoms of ADHD be consistent with an individual’s developmental level. The SNAP-IV was designed for use with typically developing (i.e., non-clinical) school-aged children without serious comorbid conditions (Swanson et al., 2012). The average child in the current sample was chronologically young, had some degree of language delay, and was not attending a mainstream school. Some of the SNAP-IV items, especially those indicating inattention (e.g., “Avoids tasks requiring sustained mental effort”), may not have been relevant to children not yet enrolled in a schooling system where such behaviors are typically expected. In other words, the behaviors indicated by the items (e.g., sustaining attention, modulating verbal activity) may not have been appropriate to expect of a child, taking into account their developmental age and the presence of specific cognitive deficits. The SNAP-IV may therefore be less useful as a measure of ADHD for young children with developmental delay. Notwithstanding the somewhat supportive results of the CFA (especially given the significant differences between the ‘target’ sample and the current sample), the clinical context in which the tool is being administered has important implications for the interpretation of items and responses.

The framework for the current study’s analyses was based on a previous study by Yerys and colleagues (2017). Although the two study samples were very different in terms of chronological age, developmental diagnoses, and cognitive functioning, the findings were relatively consistent. Notably, both studies found that existing ADHD rating scales may not adequately tap into the latent constructs of inattention and hyperactively respectively in children with NDDs. Two items (“Does not listen when spoken to directly” and “distracted by extraneous stimuli”) seemed to be poor indicators of inattention in both samples. In addition, both studies demonstrated that the ADHD scales had good construct validity when correlated with internalizing and externalizing behaviours respectively. Overall, this study upholds Yerys and colleagues’ conclusion that standard ADHD rating scales may not detect ADHD symptoms with sufficient precision in children with neurodevelopmental disorders.

## Limitations

This study had three major limitations. Most of the children in this sample were chronologically young, were diagnosed with ASD or ID, and had some degree of cognitive or language delay. Although the sample was representative of children attending neurodevelopmental clinics in sub-Saharan Africa (Bakare & Munir, 2011; Springer et al., 2013), these results cannot be generalized to NDD populations with different proportions of severe and non-verbal cases. A larger sample of children with NDDs, stratified by diagnosis, chronological age, and degree of language delay would have allowed a more thorough evaluation of the validity of the SNAP-IV in the NDD population. Another important limitation was the relatively small sample size. CFA techniques often require large sample sizes to ensure precision and generalizability of the results (Kline, 2016). Chi-square tests, fit indices, parameter estimates, modification indices, and standardized residuals are all sensitive to sample size (Kyriazos, 2018). Larger samples are often recommended, especially when models are complex, data are not normally distributed, and when data are missing (Kline, 2016). Although a sample size of at is least 200 is generally considered sufficient, a larger sample would likely produce a more stable solution. Finally, including a variety of behavioral measures with different measurement methods would have likely provided more accurate and reliable estimates of convergent and discriminant validity of the SNAP-IV.

## Summary and Conclusion

Clinicians working in low-resource settings in the Western Cape often administer the SNAP-IV to evaluate ADHD symptoms in children with NDDs. Data derived from the SNAP-IV have important implications for referral and the provision of interventions for comorbid ADHD. However, little is known about its validity in the NDD population, especially in children with moderate to severe ID and language delay as part of their phenotype. The primary aim of this study was to evaluate the validity of the SNAP-IV in a sample of children with NDDs using CFA techniques. The results of the CFA partially supported the validity of the SNAP-IV in this sample. However, there were limitations to its performance related to the clinical characteristics of the sample. The presence of developmental delay and specific cognitive deficits associated with one or more NDDs likely influenced ratings of target ADHD behaviors. Importantly, some SNAP-IV items may be unsuitable measures of hyperactivity-impulsivity in children without functional speech. Other items, especially those measuring inattention, may be less relevant to children not yet enrolled in a formal schooling system. Additional studies are needed to determine whether existing DSM-based ADHD rating scales are able to capture inattentive and hyperactive/impulsive behaviors in the NDD population with sufficient precision. Refinement and rewording some SNAP-IV items may be warranted to improve measurement accuracy.
